# Chromosome 1q alterations in transplant-eligible multiple myeloma: clinical impact of copy number and co-occurring high-risk cytogenetic features

**DOI:** 10.3389/fonc.2026.1896152

**Published:** 2026-08-17

**Authors:** Irene Strassl, Daniel Bindeus, Jonathan Burghofer, Martin Erdel, Veronika Buxhofer-Ausch, Sigrid Machherndl-Spandl, Olga Saini, Dagmar Wipplinger, Emine Kaynak, Robert Milanov, Natalia Rotter, Petra Hasengruber, Lorenz Mair, Alexander Nikoloudis, Ansgar Weltermann, Holger Rumpold, Johannes Clausen

**Affiliations:** 1 Department of Internal Medicine I: Hematology with Stem Cell Transplantation, Hemostaseology and Medical Oncology, Ordensklinikum Linz, Linz, Austria; 2 Medical Faculty, Johannes Kepler University Linz, Linz, Austria; 3 Laboratory for Molecular Genetics Diagnostics, Ordensklinikum Linz, Linz, Austria

**Keywords:** 1q alterations, 1q amplification, 1q gain, cytogenetics, multiple myeloma

## Abstract

**Introduction:**

Chromosome 1q abnormalities are among the most frequent cytogenetic alterations in multiple myeloma (MM), yet their prognostic relevance has been reported inconsistently and varies across current risk stratification models.

**Methods:**

We conducted a retrospective single-center study to evaluate the clinical impact of 1q abnormalities in 140 patients with newly diagnosed MM undergoing autologous stem cell transplantation (ASCT).

**Results:**

Fluorescence *in situ* hybridization at diagnosis identified 1q alterations in 40% of patients, including 1q gain (3 copies; 29.3%) and amplification (≥4 copies; 10.7%). Patients with 1q amplification had significantly shorter progression-free survival (PFS) compared to those without 1q abnormalities, whereas 1q gain was not associated with inferior outcomes. Notably, patients with co-occurring high-risk cytogenetic features (“double-hit”) had the poorest outcomes, while isolated 1q alterations did not independently impact survival. Despite achieving deeper responses both prior to and at day 100 after ASCT, patients with 1q gain/amplification did not experience improved long-term outcomes, underscoring the limited prognostic value of response assessment at a single time point. In multivariable analyses, established risk factors, including ISS stage III and IMWG high-risk cytogenetics, remained independently associated with PFS and overall survival (OS), whereas 1q abnormalities did not, although the limited number of patients with 1q amplification warrants cautious interpretation. Maintenance therapy was associated with improved PFS but not OS.

**Conclusion:**

In conclusion, the prognostic relevance of chromosome 1q alterations in transplant-eligible newly diagnosed MM appears to be primarily driven by copy number burden and co-occurrence with other high-risk lesions rather than by their isolated presence. These findings support the integration of 1q abnormalities into composite risk models to improve risk stratification.

## 1 Introduction

Multiple myeloma (MM) is a biologically heterogeneous plasma cell malignancy characterized by a wide spectrum of clinical outcomes. Despite substantial therapeutic advances over the past decade, disease relapse remains inevitable for a considerable proportion of patients (1, 2). Consequently, accurate risk stratification has become a cornerstone of contemporary MM management, enabling the identification of patients at increased risk of early progression and inferior survival and supporting the development of risk-adapted treatment strategies (3–6).

Among established high-risk cytogenetic abnormalities, deletion 17p and translocations such as t(4,14) and t(14,16) have consistently been associated with poor prognosis and have long been incorporated into staging and risk stratification systems (5). In contrast, the prognostic relevance of abnormalities involving the long arm of chromosome 1, particularly gain or amplification of 1q21, remains a matter of ongoing debate (3, 7). Chromosome 1q alterations are among the most frequent cytogenetic abnormalities in MM, occurring in approximately 40% of newly diagnosed patients, and are thought to promote disease progression through increased copy number of genes involved in proliferation, survival, and drug resistance (1, 8, 9).

Several studies have suggested that the adverse prognostic impact of 1q abnormalities may depend on the level of copy number alteration, with amplification (defined as ≥4 copies) conferring a worse prognosis than simple gain (3 copies) (10–12). However, accumulating evidence indicates that the clinical relevance of 1q abnormalities may be driven less by their isolated presence but rather by their co-occurrence with other high-risk cytogenetic features (13, 14). In line with this concept, recent efforts to refine risk stratification in MM increasingly emphasize cumulative or “double-hit” biology, where the presence of multiple adverse cytogenetic abnormalities identifies a subgroup of patients with particularly poor outcomes (3, 13). In this context, 1q alterations have been incorporated as modifiers of risk when occurring alongside other high-risk lesions (2, 3, 15).

In this study, we analyzed the clinical relevance of chromosome 1q abnormalities in patients with newly diagnosed multiple myeloma (NDMM) undergoing autologous stem cell transplantation (ASCT). Specifically, we aimed to evaluate the prognostic impact of 1q alterations and to determine whether their effect is primarily driven by copy number burden and/or by their co-occurrence with additional high-risk cytogenetic features.

## 2 Material and methods

### 2.1 Study design and patients

This retrospective single-center cohort study included consecutive adult patients with NDMM who underwent ASCT as part of first-line therapy at Ordensklinikum Linz Elisabethinen, Linz, Austria, between January 1, 2015 and December 31, 2020. Clinical and laboratory data were extracted from electronic medical records. Patients were required to have available cytogenetic data at diagnosis. Data were censored at the cut-off date of December 31, 2023. The study was conducted in accordance with the Declaration of Helsinki and approved by the local institutional review board (EK-Nr. 1169/2024).

### 2.2 Cytogenetic assessment and risk stratification

Interphase fluorescence *in situ* hybridization (iFISH) was performed at diagnosis on unselected bone marrow aspirate cells according to institutional standards. Only specimens containing ≥20% plasma cells, as determined by multiparameter flow cytometry, were included for analysis. FISH was performed according to the manufacturer’s instructions using commercially available XL probes (MetaSystems, Altlussheim, Germany): CDKN2C/CKS1B dual-color probe for detection of 1q21.3 copy number abnormalities, TP53/NF1 dual-color probe for 17p13.1 deletion, IGH break-apart probe (14q32.33), IGH/FGFR3 dual-color dual-fusion probe for t(4,14)(p16;q32), and IGH/MAF dual-color dual-fusion probe for t(14,16)(q32;q23). Testing for t(14,20)(q32;q12) was not routinely performed, as cytogenetic risk assessment followed the Revised International Staging System (R-ISS), introduced in 2015, which did not include this translocation among high-risk abnormalities (5). At least 200 interphase nuclei were evaluated for each probe set. FISH results were interpreted using validated laboratory-specific cut-off values (16). A threshold of 20% positive nuclei was applied for detection of 17p13.1 deletion, 5% for detection of 1q abnormalities, whereas a threshold of 3% was used for recurrent IGH rearrangements, including t(4,14), t(14,16), and abnormalities detected by the IGH break-apart probe.

Chromosome 1q alterations were categorized as gain (3 copies) or amplification (≥4 copies). Additional high-risk cytogenetic abnormalities were defined according to International Myeloma Working Group (IMWG) criteria (5) and included t(4,14), t(14,16), and deletion 17p. Patients were stratified into cytogenetic risk groups based on the presence of single IMWG cytogenetic high-risk features, 1q abnormalities summarizing 1q gain and amplification (1q gain/amp), and “double-hit” disease, defined as the co-occurrence of a 1q abnormality with at least one additional IMWG high-risk lesion.

### 2.3 Treatment and response assessment

Induction, conditioning, and post-transplant therapies (including consolidation and maintenance therapies) were administered according to institutional practice and physician discretion. Response to therapy was assessed according to IMWG response criteria (17). Depth of response was evaluated both prior to ASCT and at day 100 after transplantation, as well as best response achieved during follow-up.

### 2.4 Endpoints

The primary endpoint was progression-free survival (PFS), defined as the time from first ASCT to disease progression or death from any cause. Secondary endpoints included overall survival (OS), defined as the time from first ASCT to death from any cause, overall response rate (ORR), depth of response, and non-relapse mortality (NRM). NRM was analyzed in a competing risks framework, with disease progression or relapse treated as a competing event. Patients without events were censored at last follow-up.

### 2.5 Statistical analysis

Categorical variables were compared using the chi-square or Fisher’s exact test, and continuous variables using the Mann–Whitney U test as appropriate. Median follow-up was estimated using the reverse Kaplan–Meier method. Survival outcomes were estimated using the Kaplan–Meier method and compared using the log-rank test. Hazard ratios (HRs) and 95% confidence intervals (CIs) were calculated using Cox proportional hazards models. Multivariable Cox regression analyses were performed to identify independent prognostic factors for PFS and OS. Variables included in the models were selected based on clinical relevance and univariable analyses. Response status prior to ASCT was not included as a covariate in standard Cox models due to violation of the proportional hazards assumption; instead, stratified analyses were applied. To account for time-dependent treatment effects and avoid immortal time bias, the impact of maintenance therapy was analyzed using time-dependent Cox regression models. A two-sided p-value <0.05 was considered statistically significant. Statistical analyses were performed using R version 4.5.2 (R Foundation for Statistical Computing, Vienna, Austria).

## 3 Results

### 3.1 Baseline and treatment characteristics

The study included 140 patients with NDMM who underwent ASCT as part of frontline therapy between January 1, 2015 and December 31, 2020 at Ordensklinikum Linz Elisabethinen. Of these patients, 56 (40.0%) had 1q alterations, either gain (3 copies; 29.3%) or amplification (≥4 copies; 10.7%) of 1q21. Baseline and treatment characteristics of patients with and without 1q gain/amp were largely comparable, except for co-occurrence of t(4,14), which was more frequent in patients with 1q gain/amp (23.2% vs 6.0%; p<0.01), and utilization of tandem ASCT, which was performed in 19.6% of patients with 1q gain/amp and 6.0% of patients without 1q gain/amp (p=0.02) (**Table 1**; see **Supplementary Tables 1**, **2** for baseline characteristics stratified by 1q gain and 1q amplification). Twenty-eight percent of patients had an International Staging System (ISS) stage III, LDH was elevated in 17.3%. Median time from diagnosis to ASCT was 6.3 months (range, 3.7-18.5). The most frequently administered induction regimens were triplets consisting of an immunomodulatory drug (IMiD), a proteasome inhibitor (PI) and dexamethasone in 61.4% and VCD (bortezomib/cyclophosphamide/dexamethasone) in 32.9%. The induction regimen was changed due to refractory disease in 24.3% of patients. Consolidation and maintenance therapy were administered in 40.7% and 67.9% of patients, respectively (see **Supplementary Table 3** for treatment details); the median duration of maintenance therapy was 26.9 months (range, 0.2-83.1). Most patients received lenalidomide maintenance (85.3%). Only three patients did not receive further therapy following first relapse after ASCT (3/83 patients); the median number of subsequent lines of therapy was two (range, 0-11).

**Table 1:** Baseline and treatment characteristics according to 1q status .

Characteristic	Total (n=140)	1q normal (n=84)	1q gain/amp (n=56)	p-value
**Median age at ASCT, years (range)**	61 (37-74)	62 (37-73)	61 (38-74)	0.99
**Median time from diagnosis to ASCT, months (range)**	6.3 (3.7-18.5)	6.5 (3.7-18.2)	6.3 (3.8-18.5)	0.65
**Sex, n (%)**				0.06
Female	56 (40.0)	28 (33.3)	28 (50.0)	
Male	84 (60.0)	56 (66.7)	28 (50.0)	
**ISS stage, n (%)**	(n=126)	(n=76)	(n=50)	>0.99
I	51 (40.5)	31 (40.8)	20 (40.0)	
II	40 (31.7)	24 (31.6)	16 (32.0)	
III	35 (27.8)	21 (27.6)	14 (28.0)	
Unknown	14	8	6	
**LDH, n (%)**	(n=110)	(n=69)	(n=41)	0.19
Elevated	19 (17.3)	9 (13.0)	10 (24.4)	
Normal	91 (82.7)	60 (87.0)	31 (75.6)	
Unknown	30	15	15	
Cytogenetic abnormalities, n (%)				
t(4;14)	18 (12.9)	5 (6.0)	13 (23.2)	**<0.01**
t(14;16)	0 (0.0)	0 (0.0)	0 (0.0)	>0.99
del(17p)	10 (7.1)	7 (8.3)	3 (5.4)	0.74
Gain 1q	41 (29.3)	0 (0.0)	41 (73.2)	**<0.01**
Amp 1q	15 (10.7)	0 (0.0)	15 (26.8)	**<0.01**
Gain 1q + other HR CA	12 (8.6)	0 (0.0)	12 (21.4)	**<0.01**
Amp 1q + other HR CA	3 (2.1)	0 (0.0)	3 (5.4)	0.06
Induction details, n (%)				
Triplet IMiD/PI	86 (61.4)	49 (58.3)	37 (66.1)	0.38
Triplet IMiD/CD38-Ab	2 (1.4)	2 (2.4)	0 (0.0)	0.52
Quadruplet IMiD/PI/CD38-Ab	3 (2.1)	1 (1.2)	2 (3.6)	0.56
Other (mostly VCD)	49 (35.0)	32 (38.1)	17 (30.4)	0.37
No of induction regimen, n (%)				
1 regimen	96 (68.6)	57 (67.9)	39 (69.6)	0.86
>1 regimen, PD	34 (24.3)	22 (26.2)	12 (21.4)	0.55
>1 regimen, toxicity	10 (7.1)	5 (6.0)	5 (8.9)	0.52
Transplant details, n (%)				
Single ASCT	122 (87.1)	78 (92.9)	44 (78.6)	**0.02**
Tandem ASCT	16 (11.4)	5 (6.0)	11 (19.6)	**0.02**
Auto-Allo	2 (1.4)	1 (1.2)	1 (1.8)	>0.99
**Conditioning ASCT, n (%)**				0.52
Melphalan 200mg/m^2^	130 (92.9)	79 (94.0)	51 (91.1)	
Melphalan 140mg/m^2^	10 (7.1)	5 (6.0)	5 (8.9)	

1q amp, 1q amplification; ASCT, autologous stem cell transplantation; Auto-Allo, autologous followed by allogeneic transplantation; CD38-Ab, anti-CD38 monoclonal antibody; HR CA, high-risk cytogenetic abnormality; IMiD, immunomodulatory drug; ISS, International Staging System; LDH, lactate dehydrogenase; PD, progressive disease; PI, proteasome inhibitor; VCD, bortezomib/ cyclophosphamide/ dexamethasone.

Bold values indicate statistical significance (p<0.05).

### 3.2 Response to induction and autologous stem cell transplantation

The ORR before ASCT after completion of induction therapy was high at 97.6% in the overall cohort and comparable between patients with and without 1q gain/amp. However, deep responses were more frequent in patients with 1q alterations (≥CR in 32.1% vs. 16.7%; p=0.04), primarily driven by the 1q gain subgroup (**Supplementary Figure 1**).

**Figure 1:**
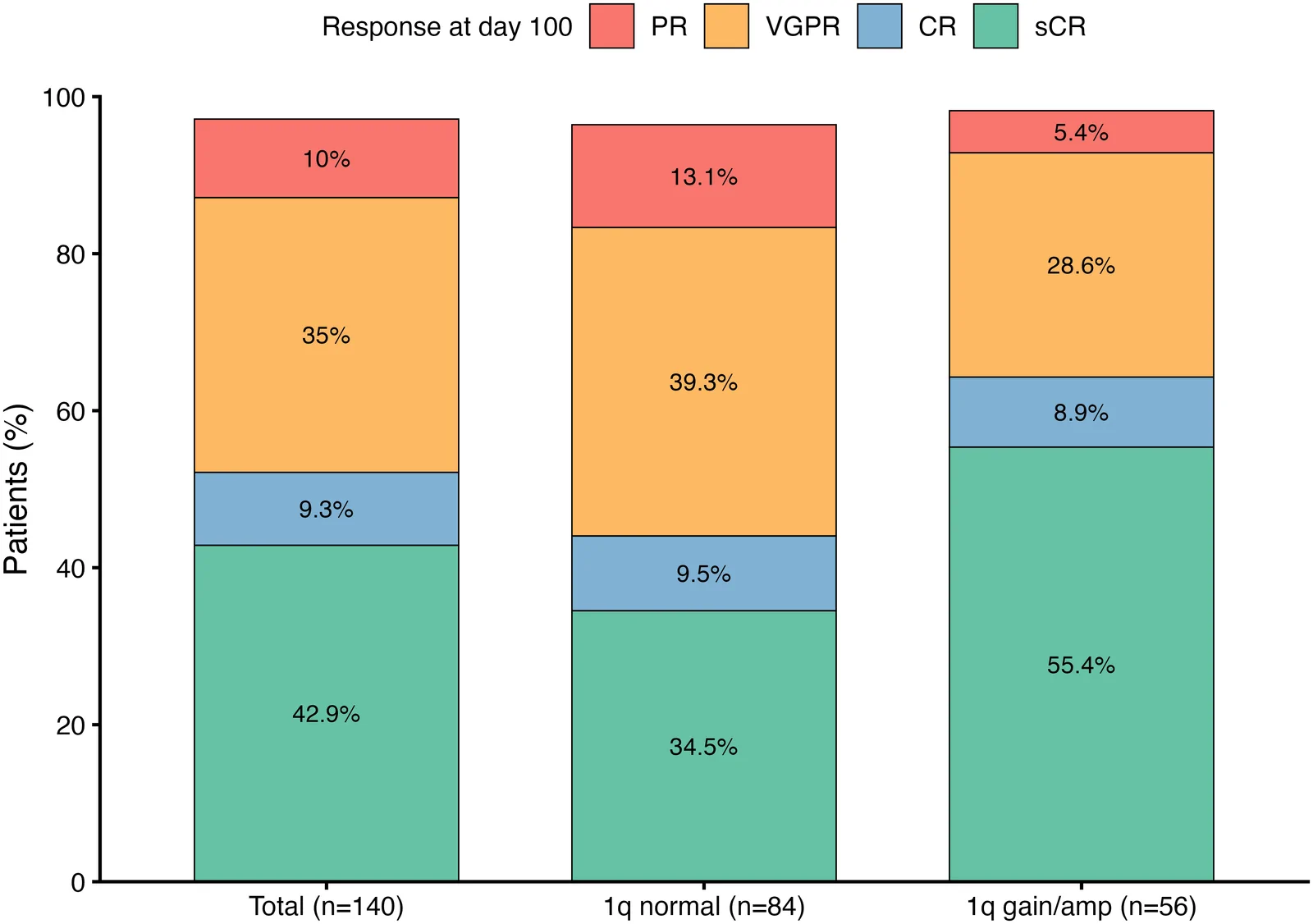
Response rates after ASCT according to 1q status. 1q amp, 1q amplification; CR, complete response; PR, partial response; sCR, stringent complete response; VGPR, very good partial response.

The 3-month ORR after ASCT was 97.1%, with no significant difference according to 1q status. A very good partial response (VGPR) or better was achieved in 92.9% of patients, and 66.4% reached a complete response (CR) or stringent CR (sCR) in the overall cohort. While ORRs were comparable, patients with 1q gain/amp showed deeper responses early after ASCT, reflected by a significantly higher CR rate at day 100 compared to patients without 1q gain/amp (64.3% vs. 44.0%, p=0.03) (**Figure 1**). This difference remained significant when considering best response at any time after ASCT (CR rate 76.8% vs. 59.5%; p=0.04).

### 3.3 Survival outcomes

Median follow-up was 59.7 months (95% CI 53.9–76.6) for PFS and 70.7 months (95% CI 64.7–80.1) for OS. Based on Kaplan–Meier estimates, median PFS was 44.4 months (95% CI 34.7–66.9) and median OS was 97.2 months (95% CI 80.2–NR) in the overall cohort (**Supplementary Figure 2**). The 60-month PFS and OS rates were 41.5% (95% CI 33.5–51.3%) and 70.2% (95% CI 62.6–78.8%), respectively. NRM was low, with a 12-month cumulative incidence of 0.7% (95% CI 0–2.1%).

**Figure 2:**
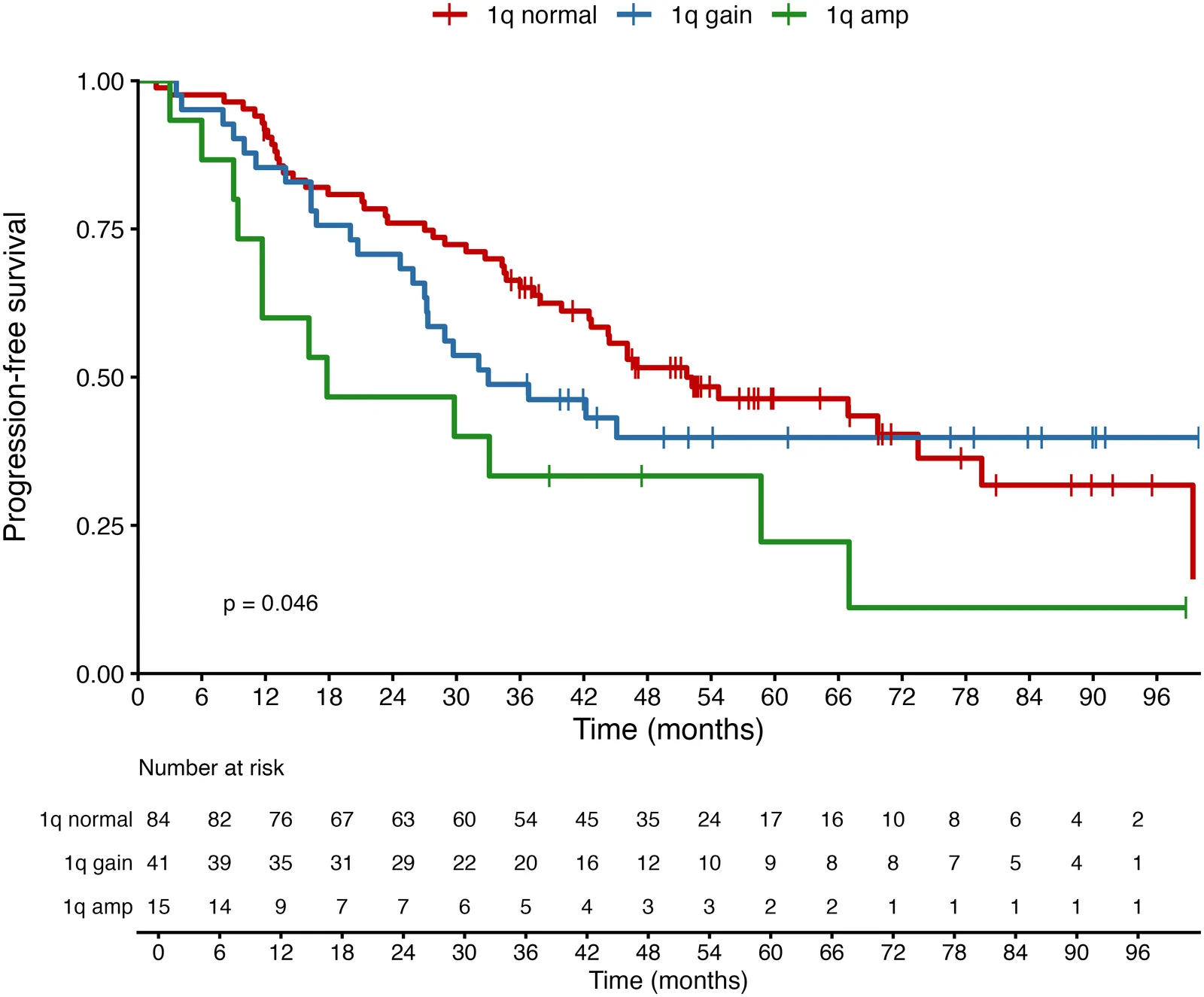
Progression-free survival according to 1q copy number status. 1q amp, 1q amplification.

Median PFS was 31.0 months (95% CI 25.9–67.0) in patients with 1q gain/amp and 51.7 months (95% CI 42.7–NR) in those without 1q abnormalities (HR 1.41, 95% CI 0.91–2.18; p=0.12) (**Supplementary Figure 3**). A separate analysis distinguishing 1q gain and 1q amplification showed that only 1q amplification was associated with significantly shorter PFS compared to patients without an alteration on chromosome 1q (median PFS 17.8 months (95% CI 11.7–NR) vs. 51.7 months (95% CI 42.7–NR); HR 2.20, 95% CI 1.17–4.16; p=0.01), whereas 1q gain was not associated with inferior PFS (median PFS 33.0 months (95% CI 27.2–NR) vs. 51.7 months (95% CI 42.7–NR); HR 1.19, 95% CI 0.73–1.96; p=0.48) (**Figure 2**). There was no significant difference in OS according to 1q status (**Supplementary Figure 4**).

### 3.4 Prognostic factors including cytogenetic risk and response

**Figure 3:**
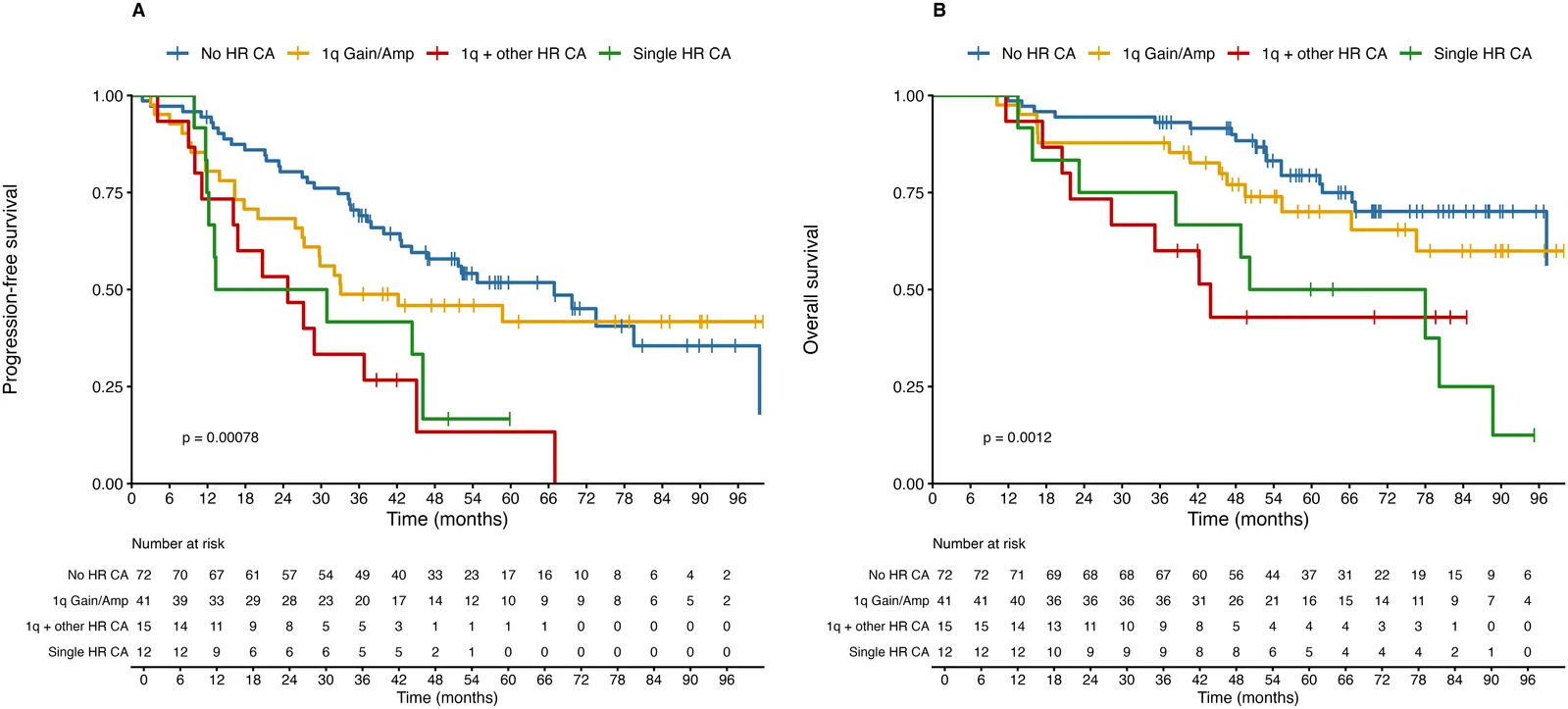
Kaplan–Meier curves for **(A)** progression-free survival and **(B)** overall survival. Survival outcomes according to cytogenetic risk groups. No HR CA, no cytogenetic high-risk abnormality incl. IMWG high-risk cytogenetic abnormalities and 1q alterations; 1q gain/amp, 1q gain or 1q amplification; 1q + other HR CA, 1q gain or amplification + IMWG high-risk cytogenetic abnormality; single HR CA, one IMWG high-risk cytogenetic abnormality excluding 1q alterations. Kaplan–Meier curves for **(A)** progression-free survival and **(B)** overall survival.

We then examined other factors that could potentially influence PFS and OS. Patients with ISS stage III had significantly shorter PFS and OS compared with ISS stage I/II (HR 2.29, 95% CI 1.41–3.71; p<0.01) (**Supplementary Figure 5**). IMWG high-risk cytogenetics were also related to poorer outcomes, particularly in conjunction with 1q alterations. Patients were categorized into no high-risk features, single-hit (t(4,14), t(14,16), or del(17p)), and double-hit (1q alteration plus another high-risk abnormality). Both single-hit (HR 2.60, 95% CI 1.29–5.27; p<0.01) and double-hit patients (HR 3.08, 95% CI 1.62–5.86; p<0.01) had significantly inferior PFS, whereas isolated 1q alterations were not associated with impaired PFS (HR 1.28, 95% CI 0.76–2.16; p=0.35) (**Figure 3**). Similar patterns were observed for OS.

No significant differences in PFS or OS were observed based on LDH levels at MM diagnosis (elevated vs. normal), number of ASCTs (single vs. tandem ASCT), or the number of induction regimens (one vs. more than one due to insufficient response) (**Supplementary Figures 6**-**8**).

**Figure 4:**
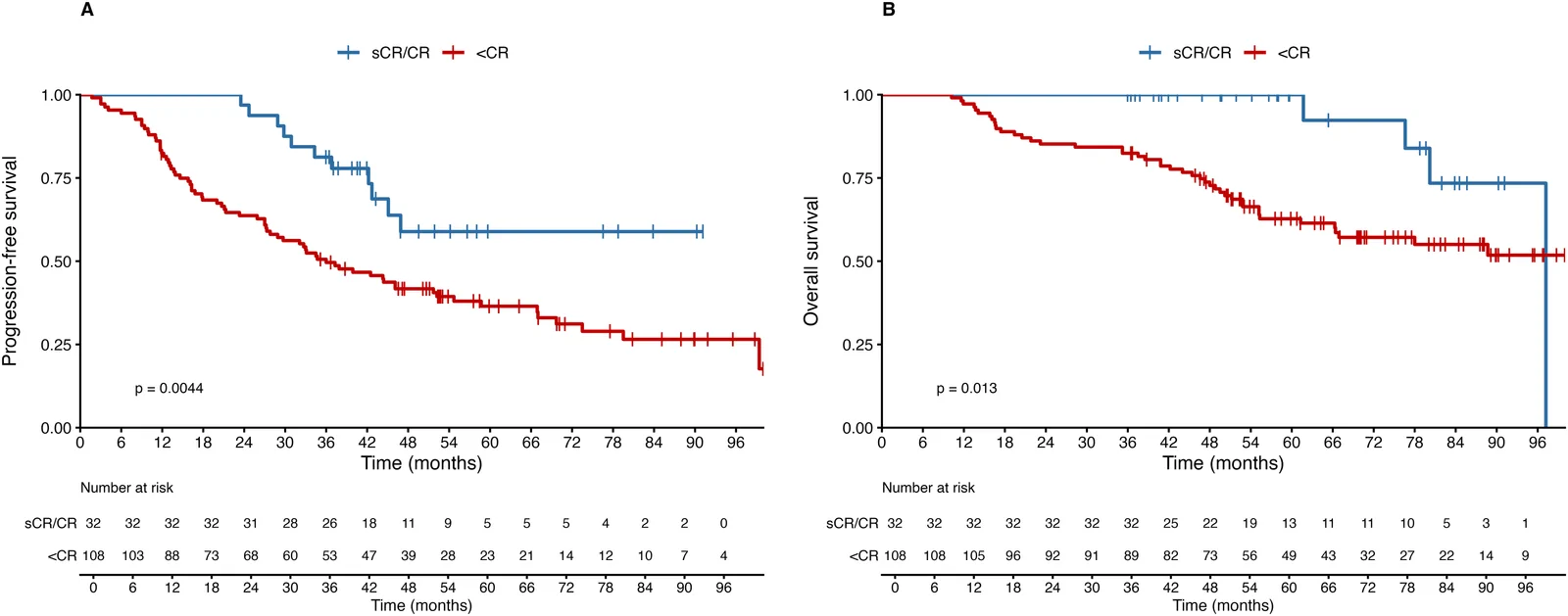
Survival outcomes according to response status prior to ASCT (≥CR vs. <CR). Kaplan–Meier curves for **(A)** progression-free survival and **(B)** overall survival. CR, complete response; sCR, stringent complete response.

Significant differences in outcomes emerged among patients according to response status prior to ASCT. Patients achieving CR before ASCT experienced significantly longer PFS and OS, whereas outcomes for all other response groups were similar. Median PFS was not reached in patients with ≥CR (95% CI 45.1–NR) compared with 36.0 months (95% CI 27.8–54.7) in patients with <CR (HR 2.45, 95% CI 1.30–4.63; p<0.01). Corresponding 60-month PFS rates were 58.9% (95% CI 42.2–82.1) versus 36.5% (95% CI 28.1–47.4), respectively. OS at 60 months was 100% versus 62.7% (95% CI 53.9–73.0) in patients with ≥CR compared to <CR (HR 3.38, 95% CI 1.21–9.41; p=0.02) (**Figure 4**). See **Supplementary Figure 9** for outcomes of the individual response groups.

### 3.5 Multivariable analyses

**Figure 5:**
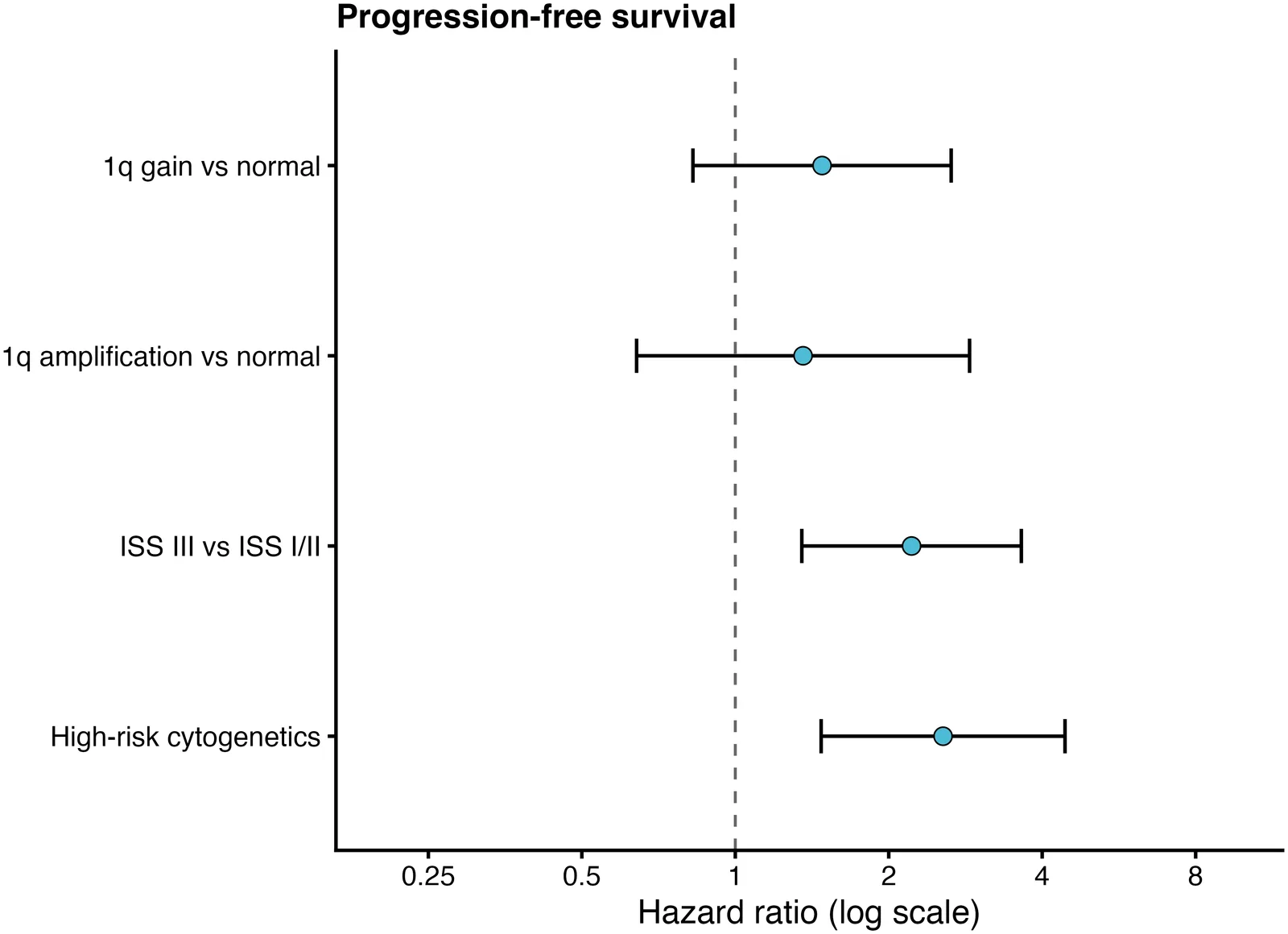
Multivariable analysis of progression-free survival. Forest plot showing hazard ratios for PFS from multivariable Cox regression analysis. High-risk cytogenetics, IMWG high-risk cytogenetic abnormality excluding 1q alterations; ISS, International Staging System.

In multivariable Cox regression stratified by response prior to ASCT (which was not included as a covariate due to violation of the proportional hazards assumption), ISS III (HR 2.22, 95% CI 1.35–3.64; p<0.01) and IMWG high-risk cytogenetics (HR 2.56, 95% CI 1.47–4.43; p<0.01) remained independently associated with inferior PFS, whereas 1q gain (HR 1.48, 95% CI 0.83–2.65; p=0.19) and 1q amplification (HR 1.36, 95% CI 0.64–2.88; p=0.43) were not (**Figure 5**). Given the limited sample size of several cytogenetic subgroups, these findings should be interpreted with caution. Similar results were observed for OS (**Supplementary Figure 10**).

The impact of maintenance therapy on outcomes was analyzed using a multivariable time-dependent Cox model to account for differences in treatment initiation and to avoid immortal time bias. In this model, maintenance therapy was associated with improved PFS (HR 0.61, 95% CI 0.37–0.99; p=0.047), but not OS (HR 1.39, 95% CI 0.71–2.70; p=0.34) (**Supplementary Figure 11**). ISS stage III and IMWG high-risk cytogenetics remained independently associated with inferior PFS (ISS: HR 2.35, 95% CI 1.42–3.88; p<0.01; IMWG high-risk: HR 2.51, 95% CI 1.42–4.47; p<0.01) and OS (ISS: HR 2.28, 95% CI 1.20–4.33; p=0.01; IMWG high-risk: HR 3.15, 95% CI 1.58–6.30; p<0.01). Neither 1q gain nor 1q amplification was independently associated with PFS (1q gain: HR 1.53, 95% CI 0.86–2.73; p=0.15; 1q amp: HR 1.36, 95% CI 0.63–2.93; p=0.43) or OS (1q gain: HR 1.83, 95% CI 0.90–3.71; p=0.09; 1q amp: HR 1.29, 95% CI 0.44–3.79; p=0.64) in these models.

## 4 Discussion

In this retrospective single-center study of transplant-eligible patients with NDMM, we examined the prognostic impact of chromosome 1q abnormalities in the context of contemporary frontline therapy including ASCT. While 1q alterations were frequent, their presence alone was not consistently associated with inferior survival outcomes.

In the overall cohort, the presence of 1q gain or amplification was not associated with significantly inferior PFS or OS. However, when distinguishing between different levels of copy number alteration, only 1q amplification (≥4 copies) was associated with significantly shorter PFS, whereas 1q gain (3 copies) was not. Importantly, our data further support the concept that the prognostic impact of 1q abnormalities is strongly influenced by their genomic context. Patients with “double-hit” high-risk cytogenetics, defined as the co-occurrence of a 1q abnormality with another high-risk lesion, had markedly inferior outcomes compared with patients without high-risk features. These findings are in line with emerging evidence that cumulative genomic risk, rather than single abnormalities, better captures disease biology and clinical behavior in multiple myeloma (3, 13, 18). Interestingly, patients with 1q alterations achieved deeper responses both prior to and early after ASCT, despite not translating into improved long-term outcomes. This apparent discrepancy may reflect an initial treatment sensitivity followed by a higher propensity for early relapse, particularly in patients with additional high-risk features. It also highlights the limitations of response assessment at single or closely spaced early time points as a surrogate for long-term outcome. Such a pattern has been described in other high-risk myeloma subgroups and underscores the limited ability of early response depth alone to capture underlying disease biology in this setting (19).

In multivariable analyses, established risk factors such as ISS stage III and IMWG high-risk cytogenetics remained independently associated with inferior PFS and OS, whereas neither 1q gain nor 1q amplification retained independent prognostic significance. However, interpretation of the multivariable analyses is limited by the small number of patients with 1q amplification (n=15), including only three patients with concomitant high-risk cytogenetic abnormalities, which may have reduced the statistical power to detect an independent effect. Accordingly, our results should not be interpreted as excluding an independent prognostic role of isolated 1q amplification. Our findings add to the heterogeneous and still evolving literature on the prognostic significance of chromosome 1q abnormalities. While some recent studies have questioned the independent prognostic impact of isolated 1q abnormalities after adjustment for co-occurring high-risk lesions, others have reported inferior outcomes in patients with 1q gain or amplification (20, 21).

The use of maintenance therapy was associated with improved PFS in a time-dependent multivariable analysis, highlighting its relevance in contemporary treatment strategies. However, this benefit did not translate into a significant OS advantage, which may reflect the availability of effective subsequent therapies, the need for longer follow-up in the context of modern treatment approaches, and the fact that maintenance therapy was ongoing in a proportion of patients at the time of data cut-off.

This study has several limitations. Its retrospective single-centre design may limit generalizability, and the sample size, particularly in the 1q amplification subgroup, was relatively small. Treatment strategies evolved over the study period and were not standardized, introducing potential confounding, including treatment allocation bias due to physician-selected intensification approaches such as the more frequent use of tandem ASCT in patients with 1q abnormalities. In addition, only a minority of patients received CD38 antibody-based induction therapy, reflecting the treatment landscape at the time and potentially limiting the applicability of our findings to current frontline approaches incorporating CD38-based quadruplet regimens. The recently updated IMS-IMWG high-risk definition (3) could not be applied, as data on del(1p) and NGS-based TP53 mutations were not available for the study period. Finally, minimal residual disease data were lacking, as they were not yet routinely available, which may have provided further insight into the observed discrepancies between response depth and survival outcomes.

Despite these limitations, our study provides additional real-world evidence supporting a risk-adapted interpretation of chromosome 1q abnormalities in transplant-eligible patients with NDMM. In particular, our findings suggest that the co-occurrence of 1q abnormalities with other high-risk cytogenetic features is an important driver of inferior outcomes, whereas isolated 1q alterations – especially 1q gain – may have a more limited prognostic impact. However, the independent prognostic relevance of isolated 1q amplification warrants further evaluation in larger cohorts.

In conclusion, our results highlight that the clinical relevance of chromosome 1q abnormalities in the setting of transplant-eligible NDMM is determined by both copy number burden and their interaction with other adverse genetic features. These findings support the integration of 1q alterations into composite risk models rather than their use as isolated markers. Future studies incorporating comprehensive genomic profiling, longitudinal disease assessment, and MRD-based evaluation are warranted to further refine risk stratification and to guide more individualized and potentially risk-adapted treatment strategies.

## Data Availability

The raw data supporting the conclusions of this article will be made available by the authors, without undue reservation.
