# Supplementary material for: Chromosome 1q alterations in transplant-eligible multiple myeloma: clinical impact of copy number and co-occurring high-risk cytogenetic features
Source: Front Oncol. 2026 Aug 17;16:1896152. doi: 10.3389/fonc.2026.1896152 (PMC13526574; doi:10.3389/fonc.2026.1896152)
Supplement: Supplementary file 1 [file DataSheet1.docx]

Supplementary Material

# 1 Supplementary Figures and Tables

## 1.1 Supplementary Figures

**Supplementary Figure 1.** **Response rates before ASCT according to 1q status.**
Abbreviations: 1q amp, 1q amplification; CR, complete response; PR, partial response; sCR, stringent complete response; VGPR, very good partial response.

**Supplementary Figure 2. Survival outcomes in the overall cohort.**
Kaplan–Meier curves for (A) progression-free survival and (B) overall survival.

**Supplementary Figure 3. Survival outcomes according to 1q status.**
Kaplan–Meier curves for (A) progression-free survival and (B) overall survival.
Abbreviations: Abbreviations: 1q amp, 1q amplification.

**Supplementary Figure 4. Overall survival according to 1q gain and 1q amplification.**
Abbreviations: Abbreviations: 1q amp, 1q amplification.

**Supplementary Figure 5. Survival outcomes according to ISS stage.**
Kaplan–Meier curves for (A) progression-free survival and (B) overall survival.
Abbreviations: Abbreviations: ISS, International Staging System.

**Supplementary Figure 6. Survival outcomes according to LDH levels at diagnosis.**
Kaplan–Meier curves for (A) progression-free survival and (B) overall survival.
Abbreviations: Abbreviations: LDH, lactate dehydrogenase.

**Supplementary Figure 7. Survival outcomes according to transplant strategy (single vs. tandem ASCT).**
Kaplan–Meier curves for (A) progression-free survival and (B) overall survival.
Abbreviations: ASCT, autologous stem cell transplantation.

**Supplementary Figure 8. Survival outcomes according to number of induction regimens.**
Kaplan–Meier curves for (A) progression-free survival and (B) overall survival.

**Supplementary Figure 9. Survival outcomes according to response categories prior to ASCT.**
Kaplan–Meier curves for (A) progression-free survival and (B) overall survival.
Abbreviations: CR, complete response; PR, partial response; sCR, stringent complete response; SD, stable disease; PD, progressive disease; VGPR, very good partial response.

**Supplementary Figure 10. Multivariable analysis of overall survival.**
Forest plot showing hazard ratios for OS from multivariable Cox regression analysis.
Abbreviations: High-risk cytogenetics, IMWG high-risk cytogenetic abnormality excluding 1q alterations; ISS, International Staging System.

**Supplementary Figure 11. Impact of maintenance therapy on survival outcomes (time-dependent analysis).**
(A) Progression-free survival and (B) overall survival according to maintenance therapy analyzed using time-dependent Cox regression models.
Abbreviations: High-risk cytogenetics, IMWG high-risk cytogenetic abnormality excluding 1q alterations; ISS, International Staging System.

## 1.2 Supplementary Tables

**Supplementary Table 1: Baseline and treatment characteristics according to 1q gain.**

| **Characteristic** | **Total**  **(n=140)** | **1q normal**  **(n=84)** | **1q gain**  **(n=41)** | **p-value** |
| --- | --- | --- | --- | --- |
| **Median age at ASCT, years (range)** | 61 (37-74) | 62 (37-73) | 62 (42-74) | 0.52 |
| **Median time from diagnosis to ASCT, months (range)** | 6.3 (3.7-18.5) | 6.5 (3.7-18.2) | 6.3 (3.8-18.5) | 0.86 |
| **Sex, n (%)**  Female  Male | 56 (40.0)  84 (60.0) | 28 (33.3)  56 (66.7) | 18 (43.9)  23 (56.1) | 0.32 |
| **ISS stage, n (%)**  I  II  III  Unknown | (n=126)  51 (40.5)  40 (31.7)  35 (27.8)  14 | (n=76)  31 (40.8)  24 (31.6)  21 (27.6)  8 | (n=38)  16 (42.1)  11 (28.9)  11 (28.9)  3 | >0.99  0.83  >0.99 |
| **LDH, n (%)**  Elevated  Normal  Unknown | (n=110)  19 (17.3)  91 (82.7)  30 | (n=69)  9 (13.0)  60 (87.0)  15 | (n=28)  6 (21.4)  22 (78.6)  13 | 0.36 |
| **Cytogenetic abnormalities, n (%)**  t(4;14)  t(14;16)  del(17p)  Gain 1q  Amp 1q  Gain 1q + other HR CA  Amp 1q + other HR CA | 18 (12.9)  0 (0.0)  10 (7.1)  41 (29.3)  15 (10.7)  12 (8.6)  3 (2.1) | 5 (6.0)  0 (0.0)  7 (8.3)  0 (0.0)  0 (0.0)  0 (0.0)  0 (0.0) | 10 (24.4)  0 (0.0)  3 (7.3)  41 (100.0)  0 (0.0)  12 (29.3)  0 (0.0) | **<0.01**  >0.99  >0.99  **<0.01**  >0.99  **<0.01**  >0.99 |
| **Induction details, n (%)**  Triplet IMiD/PI  Triplet IMiD/CD38-Ab  Quadruplet IMiD/PI/CD38-Ab  Other (mostly VCD) | 86 (61.4)  2 (1.4)  3 (2.1)  49 (35.0) | 49 (58.3)  2 (2.4)  1 (1.2)  32 (38.1) | 27 (65.9)  0 (0.0)  1 (2.4)  13 (31.7) | 0.44  >0.99  0.55  0.55 |
| **No of induction regimen, n (%)**  1 regimen  >1 regimen, PD  >1 regimen, toxicity | 96 (68.6)  34 (24.3)  10 (7.1) | 57 (67.9)  22 (26.2)  5 (6.0) | 33 (80.5)  6 (14.6)  2 (4.9) | 0.20  0.17  >0.99 |
| **Transplant details, n (%)**  Single ASCT  Tandem ASCT  Auto-Allo | 122 (87.1)  16 (11.4)  2 (1.4) | 78 (92.9)  5 (6.0)  1 (1.2) | 34 (82.9)  7 (17.1)  0 (0.0) | 0.12  0.06  >0.99 |
| **Conditioning ASCT, n (%)**  Melphalan 200mg/m^2^  Melphalan 140mg/m^2^ | 130 (92.9)  10 (7.1) | 79 (94.0)  5 (6.0) | 37 (90.2)  1 (6.7) | 0.47 |

ASCT, autologous stem cell transplantation; Auto-Allo, autologous followed by allogeneic transplantation; CD38-Ab, anti-CD38 monoclonal antibody; HR CA, high-risk cytogenetic abnormality; IMiD, immunomodulatory drug; ISS, International Staging System; LDH, lactate dehydrogenase; PD, progressive disease; PI, proteasome inhibitor; VCD, bortezomib/ cyclophosphamide/ dexamethasone

**Supplementary Table 2: Baseline and treatment characteristics according to 1q amplification.**

| **Characteristic** | **Total**  **(n=140)** | **1q normal**  **(n=84)** | **1q amp**  **(n=15)** | **p-value** |
| --- | --- | --- | --- | --- |
| **Median age at ASCT, years (range)** | 61 (37-74) | 62 (37-73) | 57 (38-70) | 0.22 |
| **Median time from diagnosis to ASCT, months (range)** | 6.3 (3.7-18.5) | 6.5 (3.7-18.2) | 7.3 (4.7-17.5) | 0.17 |
| **Sex, n (%)**  Female  Male | 56 (40.0)  84 (60.0) | 28 (33.3)  56 (66.7) | 10 (66.7)  5 (33.3) | **0.02** |
| **ISS stage, n (%)**  I  II  III  Unknown | (n=126)  51 (40.5)  40 (31.7)  35 (27.8)  14 | (n=76)  31 (40.8)  24 (31.6)  21 (27.6)  8 | (n=12)  4 (33.3)  5 (41.7)  3 (25.0)  3 | 0.76  0.52  >0.99 |
| **LDH, n (%)**  Elevated  Normal  Unknown | (n=110)  19 (17.3)  91 (82.7)  30 | (n=69)  9 (13.0)  60 (87.0)  15 | (n=13)  4 (30.8)  9 (69.2)  2 | 0.21 |
| **Cytogenetic abnormalities, n (%)**  t(4;14)  t(14;16)  del(17p)  Gain 1q  Amp 1q  Gain 1q + other HR CA  Amp 1q + other HR CA | 18 (12.9)  0 (0.0)  10 (7.1)  41 (29.3)  15 (10.7)  12 (8.6)  3 (2.1) | 5 (6.0)  0 (0.0)  7 (8.3)  0 (0.0)  0 (0.0)  0 (0.0)  0 (0.0) | 3 (20.0)  0 (0.0)  0 (0.0)  0 (0.0)  15 (100.0)  0 (0.0)  1 (6.7) | 0.10  >0.99  0.59  >0.99  **<0.01**  >0.99  **<0.01** |
| **Induction details, n (%)**  Triplet IMiD/PI  Triplet IMiD/CD38-Ab  Quadruplet IMiD/PI/CD38-Ab  Other (mostly VCD) | 86 (61.4)  2 (1.4)  3 (2.1)  49 (35.0) | 49 (58.3)  2 (2.4)  1 (1.2)  32 (38.1) | 10 (66.7)  0 (0.0)  1 (6.7)  4 (26.7) | 0.78  >0.99  0.28  0.56 |
| **No of induction regimen, n (%)**  1 regimen  >1 regimen, PD  >1 regimen, toxicity | 96 (68.6)  34 (24.3)  10 (7.1) | 57 (67.9)  22 (26.2)  5 (6.0) | 6 (40.0)  6 (40.0)  3 (20.0) | **0.05**  0.35  0.10 |
| **Transplant details, n (%)**  Single ASCT  Tandem ASCT  Auto-Allo | 122 (87.1)  16 (11.4)  2 (1.4) | 78 (92.9)  5 (6.0)  1 (1.2) | 10 (66.7)  4 (26.7)  1 (6.7) | **0.01**  **0.03**  0.28 |
| **Conditioning ASCT, n (%)**  Melphalan 200mg/m^2^  Melphalan 140mg/m^2^ | 130 (92.9)  10 (7.1) | 79 (94.0)  5 (6.0) | 14 (93.3)  1 (6.7) | >0.99 |

ASCT, autologous stem cell transplantation; Auto-Allo, autologous followed by allogeneic transplantation; CD38-Ab, anti-CD38 monoclonal antibody; HR CA, high-risk cytogenetic abnormality; IMiD, immunomodulatory drug; ISS, International Staging System; LDH, lactate dehydrogenase; PD, progressive disease; PI, proteasome inhibitor; VCD, bortezomib/ cyclophosphamide/ dexamethasone

**Supplementary Table 3: Post-transplant treatment and subsequent therapy according to 1q status.**

| **Characteristic** | **Total**  **(n=140)** | **1q normal**  **(n=84)** | **1q gain/amp**  **(n=56)** | **p-value** |
| --- | --- | --- | --- | --- |
| **Consolidation therapy** | 57 (40.7) | 35 (41.7) | 22 (39.3) | 0.86 |
| **Maintenance therapy** | 95 (67.9) | 57 (67.9) | 38 (67.9) | >0.99 |
| **Maintenance regimen**  Lenalidomide  Pomalidomide  Bortezomib  Ixazomib | (n=95)  81 (85.3)  8 (8.4)  5 (5.3)  1 (1.1) | (n=57)  46 (80.7)  6 (10.5)  4 (7.0)  1 (1.8) | (n=38)  35 (92.1)  2 (5.3)  1 (2.6)  0 (0.0) | 0.15  0.47  0.65  >0.99 |
| **Median duration of maintenance therapy, months (range)** | (n=95)  26.9 (0.2-83.1) | (n=57)  32.0 (0.2-83.1) | (n=38)  20.6 (1.2-68.4) | 0.33 |
| **Reason maintenance end**  Progressive disease  Toxicity  Other  Ongoing at data cut | (n=95)  36 (37.9)  18 (18.9)  13 (13.7)  28 (29.5) | (n=57)  17 (29.8)  11 (19.3)  10 (17.5)  19 (33.3) | (n=38)  19 (50.0)  7 (18.4)  3 (7.9)  9 (23.7) | 0.05  >0.99  0.23  0.36 |
| **Median subsequent lines of therapy following first relapse, number (range)** | (n=83)  2 (0-11) | (n=47)  2 (0-8) | (n=36)  3 (1-11) | 0.34 |
| **Second ASCT at relapse, n (%)** | 14 (10.0) | 9 (10.7) | 5 (8.9) | 0.78 |
| **AlloSCT at relapse, n (%)** | 16 (11.4) | 6 (7.1) | 10 (17.9) | 0.06 |

1q amp, 1q amplification; ASCT, autologous stem cell transplantation; AlloSCT, allogeneic stem cell transplantation.
